# Striatal cholinergic interneurons in isolated generalized dystonia—rationale and perspectives for stem cell-derived cellular models

**DOI:** 10.3389/fncel.2014.00205

**Published:** 2014-07-28

**Authors:** Philipp Capetian, Martje Gesine Pauly, Luis Manuel Azmitia, Christine Klein

**Affiliations:** ^1^Institute of Neurogenetics, University of LübeckLübeck, Germany; ^2^Laboratory of Stereotaxy and Interventional Neuroscience, Department of Stereotactic and Functional Neuroscience, University Medical Center, FreiburgGermany

**Keywords:** striatal interneurons, development, dystonia, disease modeling, induced pluripotent neurons, differentiation

## Abstract

Interneurons comprise a minority of the striatal neuronal population of roughly 5%. However, this heterogeneous population is of particular interest as it fulfills an important relay function in modulating the output of the only type of striatal projection neurons, i.e., the medium spiny neuron (MSN).One subtype of this heterogenous group, the cholinergic interneuron, is of particular scientific interest as there is a relevant body of evidence from animal models supporting its special significance in the disease process. The development of protocols for directed differentiation of human pluripotent stem cells (PSC) into striatal interneurons provides a unique opportunity to derive *in vitro* those cell types that are most severely affected in dystonia.In this review we first aim to give a concise overview about the normal function of striatal interneurons and their dysfunction in dystonia in order to identify the most relevant interneuronal subtype for the pathogenesis of dystonia. Secondly we demonstrate how knowledge about the embryonic development of striatal interneurons is of particular help for the development of differentiation protocols from PSC and by this depict potential ways of deriving *in vitro* disease models of dystonia. We furthermore address the question as to whether cell replacement therapies might represent a beneficial approach for the treatment of dystonia.

## Introduction

Dystonias are a heterogeneous group of movement disorders characterized by sustained or intermittent muscle contractions causing abnormal, often repetitive, movements, postures, or both. Dystonic movements are typically patterned and twisting and may be tremulous. Dystonia is often initiated or worsened by voluntary action and associated with overflow muscle activation (Albanese et al., 2013). Distribution of dystonia ranges from focal (one body region), segmental (two or more adjacent regions) to generalized forms (Geyer and Bressman, 2006). While complex dystonias arise from either brain damage or pharmacological/toxicological alterations, isolated dystonias have either no evident cause (previously referred to as idiopathic dystonias) or are due to inherited or spontaneous mutations in the genome (familial dystonias). In contrast, combined dystonias show additional symptoms (such as parkinsonism or myoclonus) in addition to the dystonia (Klein et al., 2014). Isolated dystonias can be further substratified with respect to body distribution into generalized, segmental, multifocal or focal forms. In the present review, we will focus on generalized, isolated dystonia which usually starts in childhood in a limb and progresses to generalize, however usually spares the neck and face (Warner and Jarman, 1998).

Although Oppenheim already presumed an organic cause of generalized dystonia in 1911 and even identified “hereditary burden to play a major role” (Oppenheim, 1911; Klein and Fahn, 2013), the bizarre nature of some manifestations and the absence of any structural abnormalities or degeneration in the isolated dystonias led to the hypothesis of dystonia being an entirely psychogenic group of disorders (Lanska, 2010). The first described mutation for generalized isolated dystonia was a GAG deletion in the *Tor1a* gene causing DYT-TOR1A (also known as DYT1) (Ozelius et al., 1997). Although additional genes have been implicated in generalized forms of dystonia in the meantime, DYT-TOR1A accounts for roughly 40% of the generalized dystonias (Valente et al., 1998) and reaches up to 90% in Ashkenazi Jews due to a founder effect (Kramer et al., 1994).

The emergence of animal models of DYT-TOR1A shed some light on the disease mechanism of dystonias and highlighted the role of striatal cholinergic interneurons in this process (Pisani et al., 2007). The advent of induced pluripotent stem cells (iPS) as a source of patient-derived and patient-specific cell lines (Takahashi et al., 2007; Park et al., 2008) also accelerated the development of neural induction and differentiation protocols offering the potential to generate regional specified human neurons *in vitro* (Chambers et al., 2009; Crompton et al., 2013; Liu et al., 2013b), paving the way for “disease in a dish” modeling.

Since no stem cell-derived *in vitro* model of dystonias has become available to date, our main goal in this review is to provide a potential “roadmap” for achieving this goal. The development of stem cell-derived *in vitro* models of neurological diseases requires a broad perspective including knowledge about the physiological function and pathological dysfunction, as well as insight into the embryogenesis of the affected cell type, since these serve the basis for the successful generation of directed differentiation protocols and their subsequent evaluation.

When it comes to modeling of diseases with iPS, the best suited model for this purpose are inherited conditions which are based on a known gene or mutations. Since all somatic cells of the organism carry this mutation, so will the cells being reprogrammed and the derived iPS. We will limit this review on the best-characterized form of inherited dystonia, i.e., isolated generalized DYT-TOR1A and will focus on findings from the corresponding animal model and the striatal cholinergic interneuron as the cell type with the best documented functional alterations in this context.

As another possibility of employing stem cell-derived striatal interneurons, we will discuss whether cell replacement strategies might represent a beneficial approach in treating certain subgroups of dystonia patients.

## Striatal interneurons

The human striatum is composed of two morphologically but not functionally distinct parts: the putamen and the caudate ncl. It is an integral part of the basal ganglia and serves as a relay station integrating glutamatergic input from the cortex and thalamus and dopaminergic input from the substantia nigra for selection, facilitation or suppression of action and movement (Groenewegen, 2003). The majority of striatal neurons (95%) are GABAergic projection neurons, the so called medium spiny neurons (MSNs).

The remaining 5% of striatal neurons are formed by a heterogeneous population of interneurons that modulate the striatal output of the MSN. At least five classes of interneurons can be subdivided in the mammalian striatum based on size, neurotransmitter, (immuno)cytochemical, and electrophysiological properties (Kawaguchi, 1993): neurons of the first group are medium-sized, GABAergic, express the calcium-binding protein parvalbumin and show a fast spiking activity (FS). The second group is also medium-sized and GABAergic; based on its electrophysiological characteristics cells of this group are termed persistent and low-threshold spike (PLTS) cells. They express the neuropeptides somatostatin (SOM), neuropeptide Y (NPY) and the nitric oxide synthase (NOS; Figueredo-Cardenas et al., 1996). Interneurons of the third group are small- to medium-sized, GABAergic, and express the calcium-binding protein calretinin (Tepper et al., 2010). The fourth group contains the largest cells of the striatum, which are, in contrast to all other striatal neurons, cholinergic and show a long-lasting afterhyperpolarization (LA) after excitation (Kawaguchi et al., 1995). The fifth subtype is comprised of medium-sized neurons, which are also GABAergic despite their positivity for tyrosine hydroxylase (TH), electrophysiologically, the latter can be subdivided into at least four subgroups (Ibáñez-Sandoval et al., 2010).

**Figure 1:**
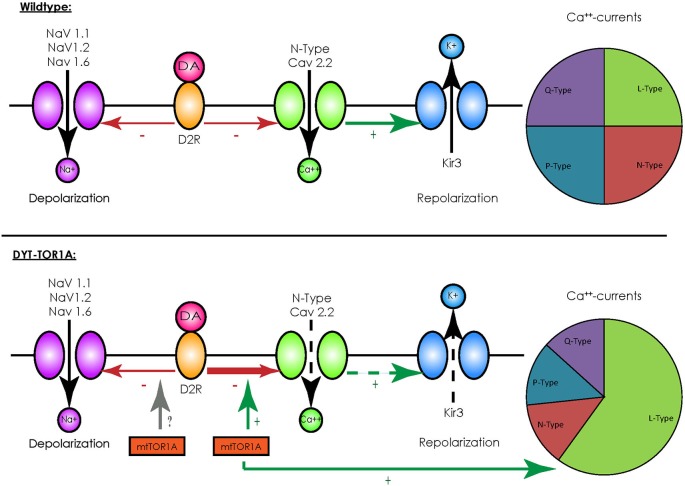
**Overview of D2-receptor coupling in striatal cholinergic interneurons**. Under physiological conditions (upper panel) binding of dopamine (DA) to D2 receptors (D2R) has an inhibitory effect on voltage-activated sodium channels (NaV 1.1, 1.2 and 1.6), which depolarize the cell membrane, and to N-type calcium channels (CaV 2.2), which activate inward-rectifying sodium channels (Kir3) and hyperpolarize the cell membrane. Although this represents two opposing effects, the net effect of D2R-activity results in a reduction of spiking and the spontaneous firing rate. In the presence of mutated TorsinA (mtTor1A) (lower panel), the inhibitory coupling of D2R to CaV 2.2 is enhanced and the amount of currents through the N-type channels in proportion to the total cellular calcium currents is increased (represented by the changes in the pie charts). This shifts the net effect of D2R towards enhanced activity of cholinergic interneurons. Whether mutant TorsinA also affects the coupling of D2R to NaVs is currently unknown.

In contrast to other striatal cells, striatal cholinergic interneurons show a spontaneous spiking acitivity, leading to the term “tonically active neurons” (TAN; Bennett and Wilson, 1999). *In vivo* recordings in primates demonstrated that salient stimuli evoked a short bursting of these neurons followed by a pause in firing, a response also elicited by conditioned stimuli after classical conditioning (Aosaki et al., 1994). These findings were detailed by *in vitro* recordings of acute rodent brain slices consisting of the cortico-striatal and thalamo-striatal connections: glutamatergic thalamo-striatal projections on cholinergic interneurons evoke the burst-pause response which suppresses transiently cortico-striatal projections on MSN, followed however by a phase of enhanced responsiveness of MSNs to cortical inputs in terms of an enhanced connectivity (Ding et al., 2010). It is therefore assumed that those interneurons mediate a “stop and no-go” response to salient stimuli followed by a redirection of attention. This response is modulated by dopaminergic nigro-striatal projections innervating the cholinergic interneurons by D2 receptors (D2R; Reynolds et al., 2004; Smith and Villalba, 2008). As G-protein-coupled receptors, D2R inhibit voltage-gated sodium channels (NaV 1.1, 1.2 and 1.6) and reduce the spontaneous spiking activity of cholinergic interneurons (Carr et al., 2003). They, however, also act in an inhibitory manner on N-type CaV 2.2 calcium channels that promote the opening of hyperpolarizing inward-rectifying potassium channels (Kir3) (Goldberg and Wilson, 2005). Under physiological conditions the resulting net effect of an activation of D2R is a reduction of spiking activity (see Figure 1). For a summary of the different interneuronal subtypes, see Table 1.

**Table 1:** **Overview of striatal interneurons**.

**No.**	**Marker**	**Neurotransmitter**	**Size**	**Electrophysiological properties**	**Assumed function**
1	PV	GABA	M	Fast spiking activity (FS)	Feed-forward inhibition from cortical afferents to MSN
2	SOM/NPY/NOS	GABA + neuropeptides	M	Persistent and low-threshold spike (PLTS)	Modulation by release of neuropeptides
3	CR	GABA	S	Unknown	Unknown
4	ChAT	Ach	L	Tonically active neurons (TAN)	Relaying thalamo-striatal and cortico-striatal input, modulated by nigrostriatal afferents
5	TH	GABA	M	Heterogenous	Modulating MSN

*Acetylcholine (Ach), choline acetyl transferase (ChAT), calretinin (CR), large size (L), medium size (M), medium spiny neuron (MSN), neuropeptide Y (NPY), nitric oxide synthase (NOS), parvalbumin (PV), small size (S), somatostatin (SOM), tyrosine hydroxylase (TH)*.

## Striatal cholinergic interneurons in DYT-TOR1A

In order to study isolated dystonias on an experimental basis, appropriate animal models are needed. Animal models of dystonia are manifold and can be divided into a toxic and a genetic category: the genetic category better reproduces molecular and neurophysiologic features, however, the motor phenotype is typically more subtle (for a complete review see Oleas et al., 2013; Wilson and Hess, 2013). Although there are many known genes, mutations in which lead to dystonia (Lohmann and Klein, 2013) animal models are largely unavailable.

As mentioned above, DYT-TOR1A dystonia is caused by a heterozygous 3-bp GAG deletion in the *Tor1a* gene (Ozelius et al., 1997). The product of the gene (TorsinA) is as a member of the AAA+ (ATPases associated with a variety of cellular activities) protein family and fulfills a widespread role in vesicle trafficking and serves as a chaperone in protein processing (Hanson and Whiteheart, 2005). The deletion of GAG results in the loss of a c-terminal glutamic acid residue and is thought to destabilize the tertiary structure of the protein acting as to a loss-of-function (Kock et al., 2006). Moreover mutant torsinA impairs the function of wildtype protein and is therefore exerting a dominant negative effect (Torres et al., 2004). The neuron-specific effect is believed to be the result of a lack of other forms of torsins, notably torsinB in neurons, that could compensate for the relative shortage of functional torsin (Jungwirth et al., 2010). Certain levels of normal torsinA are obviously crucial for the embryonic development as complete knock-outs, but also combinations of knock-out of wildtype and knock-in of mutant torsinA, resulting in neonatal lethality (Goodchild et al., 2005; Yokoi et al., 2012).

Animal models for DYT-TOR1A have been established for invertebrates (namely *C. elegans* and *D. melanogaster*) (Caldwell et al., 2003; Koh et al., 2004; Cao et al., 2005) which are suitable models for studying basic principles of the disease mechanisms on a multi-cellular level. However, the vast differences to the mammalian central nervous system limit the explanatory value for complex neuronal networks.

More relevant in terms of understanding alterations in neuronal networks in DYT-TOR1A are of course rodent models. Of these, the most relevant one is the Dyt1 ΔGAG, which consists of a heterozygous GAG deletion in the murine *TOR1A* gene, the same recurrent mutation observed in DYT-TOR1A patients. Another group of models are brain region-specific knock-outs of *TOR1A* based on the CRE-lox-system (Yokoi et al., 2008; Sciamanna et al., 2012a). These models are particularly helpful for studying the contribution of different cerebral regions to the disease process. The third group is comprised of transgenic models engineered to overexpress human mutant or wildtype torsinA driven by different promoters, such as the neuron-specific enolase (NSE; Shashidharan et al., 2005) or the constitutively active human cytomegalovirus (hCMV) promoter (Sharma et al., 2005). However, the results of this latter group of models need to be interpreted with care as observed effects could also be attributed to ectopic expression or unphysiologically high levels of torsinA.

A particular motor phenotype was found in the mouse model expressing the human mutated *TOR1A* gene under control of the NSE promoter (Shashidharan et al., 2005): transgenic animals showed enhanced motor activity in the open field test which increased significantly over time, and some animals displayed an abnormal shaking of the head from one side to the other or even tonic lateralized deviations of it. Another feature was “self clasping” of the limbs when hung from the tail. Admittedly, this phenotype differs from the pronounced generalized dystonia known from patients suffering from DYT-TOR1A, however, it must be kept in mind that—as we learned from other transgenic models of neurodegenerative diseases (like the transgenic models of Huntington’s disease (Figiel et al., 2012))—motor abnormalities in rodents often differ considerably from those seen in humans.

Despite these drawbacks, animal models have helped to identify alterations in neuronal networks possibly leading to dystonia. Their value was further emphasized by the fact that a reduction of the dopamine-binding capacity of striatal type 2 dopamine-receptors (D2R; Dang et al., 2012), as well as alterations in the cerebellothalamocortical pathways could be observed (Uluğ et al., 2011), findings already known from human mutation carriers (Asanuma et al., 2005; Carbon et al., 2010a,b). The transgenic knock-in models have been extensively characterized on an electrophysiological level and broadened our understanding of the neuronal network alterations potentially leading to dystonia: for example, recordings of MSNs in mice overexpressing mutant human torsinA showed a pronounced alteration of glutamatergic synaptic transmission. While a long-term depression (LTD) could not be elicited, long-term potentiation (LTP) was more pronounced and synaptic depotentiation (SD) to low-frequency stimuli (LFS) could not be evoked (Martella et al., 2009). These alterations could be reverted by either lowering acetylcholine levels or antagonizing muscarinic receptors of the M1-type linking these changes to altered cholinergic transmission. Recordings of striatal cholinergic interneurons did indeed reveal an altered response to thalamic stimulation, as the evoked burst-pause response was significantly shorter and followed by an abnormal spiking activity (Sciamanna et al., 2012b). As the pause response is a D2R-dependent process (Goldberg and Reynolds, 2011), a link between the already mentioned altered dopaminergic response in DYT-TOR1A and this phenomenon seems likely. Accordingly, a profound alteration of the D2R-mediated response could be demonstrated in striatal cholinergic interneurons, as their activation caused a paradoxical excitation instead the expected inhibition (Pisani et al., 2006). The cause of this alteration is an enhanced inhibitory coupling of D2R to the previously mentioned N-type Cav 2.2 channels and an increased proportion of their currents to the overall cellular calcium currents, leading to a rise in spiking activity (Figure 1). The cause of this enhanced inhibitory coupling is at the moment unclear, however, it is tempting to speculate that the regulator of G-protein signaling 9–2 (RGS 9–2), which was shown to modulate the coupling between D2R and Cav 2.2, might play a role in this regard. However, this does not answer the question whether the inhibitory coupling to the voltage-gated sodium channels (which should counterbalance this effect) is not enhanced (Cabrera-Vera et al., 2004). In addition, these results need to be interpreted with caution as the ubiquitous over expression of mutant torsinA in this model does not closely correspond to the actual situation in DYT-TOR1A. However, these findings were reproduced in a mouse model with a targeted deletion of TOR1A in cholinergic interneurons, supporting the findings that mutant torsinA exerts a dominant-negative effect on wildtype torsinA (Sciamanna et al., 2012a).

The key point is, however, how these findings explain the occurrence of the dystonic movement disorder? Current models of basal ganglia function are based on the presence of parallel distinct loops connecting the cortex with different parts of the basal ganglia and thus facilitating wanted and suppressing unwanted movement. In contrast, dystonia results from a co-contraction of muscle groups usually not activated during a particular task, a phenomenon termed “dystonic overflow” (Mink, 2003). As the striatum plays an important role in the basal ganglia circuitry, it is tempting to speculate that enhanced LTP and reduced LTD based on an increased cholinergic tone in dystonia favors the promotion of aberrant and contradicting motor commands (Sciamanna et al., 2012b), however, more experimental data will be needed to further clarify this matter.

While the role of GABAergic interneurons in DYT1 has not yet been studied in depth, one study found altered intracellular calcium dynamics in heterozygous ΔE-torsinA knock-in mice leading to a higher excitability of those cells (Iwabuchi et al., 2013).

## Embryonal development of striatal interneurons

**Figure 2:**
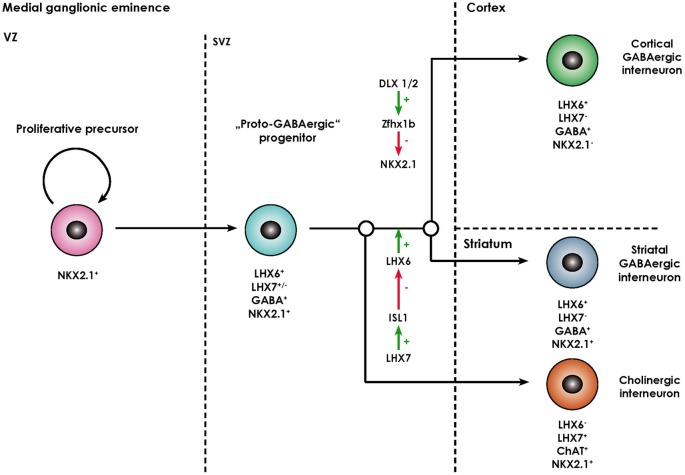
**Overview of the molecular processes involved in the specification of MGE-derived forebrain interneurons (adapted from Fragkouli et al., 2009)**. Forebrain interneurons are derived from NKX2.1+ proliferative progenitors located in the ventricular zone (VZ) of the MGE. Once they exit the cell cycle they move to the subventricular zone (SVZ) and start expressing LHX6. As they contain GABA, they are termed “proto-GABAergic”. When LHX6 expression is maintained, they either continue to express NKX2.1 and differentiate towards striatal GABAergic interneurons. Alternatively, the zinc finger homeobox gene Zfhx1b, which acts downstream of DLX1/2, represses NKX2.1 expression, in which case cortical GABAergic interneurons arise. Through ISL1, LHX7 expression leads to a repression of LHX6 and thus differentiation towards cholinergic neurons positive for choline acetyl transferase (ChAT).

The striatum (caudate nucleus and pallidum) develops from the lateral (LGE) and medial ganglionic eminence (MGE) located in the basal part of the telecephalon. While striatal projection neurons (namely MSN) arise from the lateral ganglionic eminence (Deacon et al., 1994), almost all striatal interneurons as well as cortical interneurons, cholinergic basal forebrain projection neurons, projection neurons of the globus pallidus, and telencephalic oligodendrocytes are generated in the MGE and reach their final destination either by migrating tangentially or radially (Anderson et al., 1999; Marin et al., 2000; Kessaris et al., 2006). The ventricular zone (VZ) of the MGE comprises neural progenitors expressing the NKX2.1 homeodomain protein, required for their specification (Butt et al., 2008), their patterning being in turn dependent on the morphogen sonic hedgehog (SHH; Xu et al., 2010). After exiting the cell cycle, postmitotic NKX2.1+ progenitors start migrating out of the MGE and express the LIM-homeodomain-transcription factor LHX6 (Du et al., 2008). This process is dependent on the transcription factors DLX1/2 and MASH1 (Marin et al., 2000). These immature neurons also contain GABA and share certain functional characteristics with mature GABAergic neurons, however, lack other molecular markers of specified interneuronal populations (like PV, SOM, NOS), so that they are termed proto-GABAergic (Flames and Marín, 2005). These cells are bipotential precursors being able to terminally differentiate either into the GABAergic or cholinergic lineage. Terminal differentiation into GABAergic interneurons is dependent on sustained expression of LHX6 and thus representing a sort of “default” maturation pathway (Liodis et al., 2007; Zhao et al., 2008). Differentiation into the cholinergic lineage requires expression of LHX7 (also known as L3/LHX8) which induces expression of another LIM-homeodomain-transcription factor islet-1 (ISL1), which directly downregulates LHX6 expression (Fragkouli et al., 2009). Interestingly, the cholinergic phenotype remains dependent on ongoing LHX7 expression even in postmitotically committed cholinergic neurons, as deletion of the LHX7 gene leads to a switch of phenotype to GABAergic interneurons (Lopes et al., 2012). The processes described above are summarized in Figure 2.

## Deriving striatal interneurons from pluripotent stem cells

Although animal models have been of great help in elucidating altered cellular mechanisms in dystonia and will most likely remain unsurpassed when it comes to the study of complex neural networks, obtaining human neurons is highly desirable in order to confirm findings from animal models or gaining new insights into mechanisms of the human basal ganglia. Pluripotent stem cells (PSC) can be differentiated into cells of the entire body, including neurons. iPS even allow generating patient-specific cells, sharing all possible genetic deviations and variants of the donor patient (Yamanaka, 2009).

In order to obtain a neuronal population enriched for a desired regional phenotype, it is necessary to recapitulate the *in vivo* developmental processes under *in vitro* conditions.

Patterning primitive neuroectoderm or neuroepithelial precursors in order to obtain a desired regional phenotype requires the presence of morphogens mediating the expression of downstream effectors, the most important in this respect being SHH, fibroblast growth factors (FGFs), bone morphogenetic proteins (BMPs), and agonists of the wingless/Int-1 pathway (WNT; Hébert and Fishell, 2008; Sousa and Fishell, 2010; Cho et al., 2013). Patterning neural cells *in vitro* requires the addition of morphogens in a timed manner and at appropriate concentrations. Since almost all forebrain interneurons arise from the MGE in the ventral forebrain, the addition of morphogens required for ventral forebrain specification to the cell culture should promote a corresponding phenotype. Accordingly, all published protocols utilize the strong ventralizing activity of SHH or its small molecule agonists (Bissonnette et al., 2011; Crompton et al., 2013; Germain et al., 2013; Liu et al., 2013a,b; Nicholas et al., 2013; Duan et al., 2014). In this respect, a dose dependency was demonstrated, as increasing the dose of SHH increased the number of NKX 2.1+ MGE-progenitors with doses ranging up to 1000 ng/ml (Li et al., 2009; Liu et al., 2013b). Another common component for MGE patterning is FGF8 due to its important role in ventral forebrain patterning (Storm et al., 2006). Although many published protocols include FGF8 (Bissonnette et al., 2011; Danjo et al., 2011), there are also reports omitting FGF8. As SHH concentrations used are higher in these protocols, it can be speculated that FGF8 can be replaced by high SHH concentrations (Liu et al., 2013a,b). The canonical WNT/β-catenin-pathway plays an important role in forebrain patterning both on the rostro-caudal- and ventro-dorsal axis: patterning of the dorsal telencephalon depends on activation of canonical WNT signaling (Backman et al., 2005). Conversely, cells of the ventrally localized VZ express the dickkopf proteins (a family of WNT-antagonists) (DKK; Diep et al., 2004) and combined SHH and DKK1 exposure enhances ventral telencephalic identity (Li et al., 2009; Nicoleau et al., 2013). Many protocols achieving successful MGE patterning however do not employ WNT modulation. A potential reason for this could be the fact that DKK alone has no pronounced ventralizing effect but rather potentiates SHH activity (Li et al., 2009), so that high levels of SHH alone could also be sufficient. The pronounced rostralizing effect of DKK1 (Caneparo et al., 2007) is obviously not necessary for successful patterning of *in vitro* ventral-forebrain patterning, since neural induction of PSCs leads to an anterior-dorsal identity by default (Pankratz et al., 2007).

Under optimal conditions, over 90% of the cells express NKX 2.1, thereby demonstrates MGE identity. These neurons are 45% cholinergic and 55% GABAergic (Liu et al., 2013b). This underlines the general fact that *in vitro* patterning can indeed specify the regional identity of cells in a highly effective manner, however, full specification to a single cell type (e.g., solely cholinergic) cannot be achieved in this way. One more recent protocol reported achievement of 94% forebrain cholinergic interneurons by transient overexpression of GBX1 and LHX7 (L3/LHX8) (Bissonnette et al., 2011). This example demonstrates how knowledge of molecular pathways in neural differentiation can help increase the specificity of directed differentiation beyond the scope of regional patterning.

## Rationale for cell replacement strategies in dystonia

Cell replacement strategies as potential treatment for neurodegenerative disorders have been carried out for a couple of decades with promising results in animal models but mixed outcome in clinical trials (Bjorklund and Kordower, 2013; Kim et al., 2013; Lindvall, 2013). While replacing a subpopulation of neurons seems at least in theory a feasible approach for treating neurodegenerative disorders, isolated dystonias as the DYT-TOR1A lack an overt cell loss. Accordingly, there are no reports of neural cell transplantation approaches in the current literature. Assuming the area of application for neural cell transplantation is neurodegenerative disorders with predominant affection of a confined cell type, isolated dystonias do obviously not qualify for such an approach due to the lack of neurodegeneration. On the other hand, complex dystonias frequently show too widespread a damage (Klein et al., 2014). Possibly the only potentially promising predominantly dystonic movement disorder is the combined dystonia DYT-TAF1 (X-linked dystonia parkinsonism = XDP). This disease is characterized by an adult-onset initial phase of generalized dystonia with oromandibular predominance, followed by a second predominantly hypokinetic-rigid phase (Klein et al., 2014). This disease is caused by a pronounced degeneration of MSNs inside the striosome compartment in the first stage of the disease, followed by a degeneration of MSNs inside the matrix compartment during the second phase, while relatively sparing the striatal interneurons (Goto et al., 2005; Kaji et al., 2005). Therefore, a possible neurorestaurative approach to DYT-TAF1 would indeed be the transplantation of precursors for MSNs out of the LGE, similarly as in Huntington’s disease.

## Conclusions

The absence of neurodegeneration identifies isolated dystonias as neuronal network diseases. Interneurons representing roughly 5% of the striatal neural population play an important role in modifying the output of the MSN as projection neurons and relaying thalamo-striatal and cortico-striatal inputs, especially cholinergic interneurons play an important role in integrating sensory input and redirecting attention. Using the example of DYT-TOR1A as the best studied form of isolated dystonia, we present the current knowledge as to how striatal cholinergic interneurons may play a central role in the pathophysiology of dystonia through an altered response to dopaminergic input leading to overactivity and dysfunctional gating of thalamostriatal input.

We summarized the origins and developmental processes of forebrain interneurons in combination with the molecular mechanisms that switch between GABAergic and cholinergic phenotypes.

We demonstrated on the basis of recent protocols, how knowledge of the developmental mechanisms can be employed for directed neural differentiation of PSC in order to obtain human forebrain interneurons *in vitro*. Although to date no published study employed PSC-derived striatal interneurons in order to study dystonia, this technique holds great potential for deciphering the underlying disease mechanisms of dystonia, as the necessary protocols have already been published and just await their application for *in vitro* modeling of DYT-TOR1A. We finally discuss potential neurorestaurative approaches for different subtypes of dystonias: while isolated dystonias most likely are no suitable candidates for this procedure due to the lack of neurodegeneration and complex dystonias are commonly characterized by too widespread a neural damage, transplantation of MSN precursors could be a potential field of application in XDP or DYT-TAF1 with its relatively confined degeneration of striosomes.

## Conflict of interest statement

The authors declare that the research was conducted in the absence of any commercial or financial relationships that could be construed as a potential conflict of interest.
